# Modern Diagnostic Techniques for the Assessment of Ocular Blood Flow in Myopia: Current State of Knowledge

**DOI:** 10.1155/2018/4694789

**Published:** 2018-01-21

**Authors:** Ewa Grudzińska, Monika Modrzejewska

**Affiliations:** Department of Ophthalmology, Pomeranian Medical University in Szczecin, Szczecin, Poland

## Abstract

Myopia is the most common refractive error and the subject of interest of various studies assessing ocular blood flow. Increasing refractive error and axial elongation of the eye result in the stretching and thinning of the scleral, choroid, and retinal tissues and the decrease in retinal vessel diameter, disturbing ocular blood flow. Local and systemic factors known to change ocular blood flow include glaucoma, medications and fluctuations in intraocular pressure, and metabolic parameters. Techniques and tools assessing ocular blood flow include, among others, laser Doppler flowmetry (LDF), retinal function imager (RFI), laser speckle contrast imaging (LSCI), magnetic resonance imaging (MRI), optical coherence tomography angiography (OCTA), pulsatile ocular blood flowmeter (POBF), fundus pulsation amplitude (FPA), colour Doppler imaging (CDI), and Doppler optical coherence tomography (DOCT). Many researchers consistently reported lower blood flow parameters in myopic eyes regardless of the used diagnostic method. It is unclear whether this is a primary change that causes secondary thinning of ocular tissues or quite the opposite; that is, the mechanical stretching of the eye wall reduces its thickness and causes a secondary lower demand of tissues for oxygen. This paper presents a review of studies assessing ocular blood flow in myopes.

## 1. Introduction

Myopia is the most common refractive error. Its prevalence has increased significantly over the last 50 years to about 70% of the population in developed countries. Currently, the global prevalence of myopia is estimated at about 1.4 billion people, and this figure is expected to increase to 4.7 billion by 2050 (50% of the global population) [1]. Myopia is associated with an increased risk of ophthalmic complications, such as retinal detachment and choroidal neovascularization [2]. The above medical conditions can lead to a significant deterioration of visual acuity, which creates serious economic and social problems [3]. The aetiology of myopia has not been explained in detail despite many studies on its pathogenesis. Literature data show that both genetic and environmental factors are involved in its onset [2].

High-grade myopia is associated with axial elongation of the eye, which is accompanied by changes in the structure of the sclera, choroid and retina, and retinal vasoconstriction. The thickness of the choroid in myopes is reduced compared to subjects without this refractive error [4] and at the same time correlates significantly with the axial length [5, 6]. In myopia, photoreceptors and choriocapillaris are rarefied, and the retinal pigment epithelium is diffusely depigmented [7]. In the retina, the density of the superficial and deep vascular network is reduced [8, 9]. The thinning and disappearance of the choroid and retinal structures further reduce their oxygen demand, resulting in decreased blood flow [10].

Disorders of ocular blood flow in myopia have still not been fully explained and are quite rarely described in the literature. A search of available databases (MEDLINE, Web of Science, Google Scholar) for published papers revealed several dozen publications dealing with a similar problem as discussed here. Therefore, the authors reviewed the literature with a focus on currently used diagnostic methods used for the analysis of changes in the haemodynamics of ocular blood flow in subjects with myopia.

The eyeball is supplied with blood through the ophthalmic artery (OA) and its branches. In blood flow disorders affecting the ocular artery, blood may be supplied to the eyeball by an anastomosis between the middle meningeal and the lacrimal arteries and between the angular and dorsal nasal arteries. The central retinal artery (CRA) is the first branch of the ocular artery, an anatomically terminal artery supplying blood to the inner layers of the retina. The other branches of the ophthalmic artery are short and long posterior ciliary arteries that reach the choroid and ciliary body. In addition, blood is supplied to the anterior segment by the anterior ciliary arteries [11]. The total ocular blood flow is estimated to be approx. 1 ml/min, most of which supplies the vasculature of the uvea and only 2–5% supplying the retina [12].

Ocular blood flow is affected by local factors and systemic factors, as well as autoregulation mechanisms. The latter mechanisms maintain constant blood flow in the eye despite changes in perfusion pressure which depend on fluctuations in arterial blood pressure but also in intraocular pressure or metabolic parameters [13]. Ocular factors known to affect changes in blood flow include intraocular pressure, biorheology, axial length, and diseases such as glaucoma or diabetic retinopathy. It was also found that the ocular blood flow can be altered because of blood pressure, changes in body posture, air composition, and medications [14]. Ocular blood flow is also affected by external and environmental factors such as temperature, brightness, air quality, and altitude.

## 2. Diagnostic Techniques

Currently, many methods are available for the assessment of ocular blood flow. As indicated in the literature, measurements of ocular circulation performed using different methods show similar results, but each of these methods has its limitations. Diagnostic techniques and tools include the laser Doppler flowmeter (LDF), laser Doppler velocimetry (LDV), retinal vessel analyzer (RVA), retinal function imager (RFI), laser speckle contrast imaging (LSCI), magnetic resonance imaging (MRI), optical coherence tomography angiography (OCTA), pulsatile ocular blood flowmeter (POBF), fundus pulsation amplitude (FPA), colour Doppler imaging (CDI), Doppler optical coherence tomography (DOCT), fluorescein and indocyanine angiography (FA and ICG), retinal oximetry, blue light entopic phenomenon, and infrared thermography. Using MEDLINE, Google Scholar, and Web of Science online databases, an analysis of the available blood flow studies conducted in myopic patients was performed. All techniques and findings in myopia are summarized in Table 1.

### 2.1. The Laser Doppler Flowmeter

allows for the continuous measurement of blood flow in retinal capillaries. The essence of this method is the use of a coherent laser light source and measurement of the beat frequency for light, which is partially scattered by moving blood cells and partially by surrounding tissue. In case of moving cells, the Doppler shift for source frequency is observed [15]. The readings are expressed in relative flow units, so this technique is mainly used to evaluate changes in blood flow in response to a stimulus. This method should be used, rather, to compare changes in the patient than as a comparison of different patients, because there is significant variation in the scattering of light between subjects. The device is very sensitive to head- and eye-movement artifacts, and it cannot analyze the blood flow in relation to the eye that moves along with it. Studies employing this method were conducted by Shimada et al. [16]. Measurements were taken using a Canon laser blood flowmeter, which is a combination of LDV and RVA, with an eye-tracking system for measuring flow velocity and vessel diameter, and blood flow was expressed in *μ*l/min. The study showed significant reduction of vessel diameter in eyes with myopia compared to those without refractive error. Retinal blood flow was significantly decreased in highly myopic eyes (>−8.0 diopters) compared with emmetropic eyes (±3.0 diopters) or mild myopic eyes (−3.0 to −8.0 diopters). A similar difference was observed when comparing mild myopic eyes with eyes without refractive error. The mean velocity of ocular blood flow decreased with increasing refractive error, but the correlation was not statistically significant. Shimada et al. attributed these differences to increased axial length causing mechanical stretching and thinning of the retina. Changes in the morphology of the eye structures observed by the authors may lead to straightening and decreasing vascular diameter, with a simultaneous reduction in metabolic demand of tissues for oxygen and nutrients. Another hypothesis explaining this process is the facilitated transport of oxygen into the retina via choroid of reduced density and thickness, which results in secondary retinal vasoconstriction [16]. Other researchers cooperating with Benavente-Perez observed decreased choroidal blood velocity in myopic eyes when using the above diagnostic method. They found also a higher response to hypercapnia in myopes consisting of significant increase in choroidal blood velocity [17].

### 2.2. Laser Doppler Velocimetry

The blood velocity in selected vessels can be measured using a variant of the laser Doppler velocimetry (LDV) method. Riva et al. applied successfully this method in the study of retinal arteries and veins. The essence of LDV is the observation of two collimated laser light beams, directed and focused inside the vessel in one intersection point. As a result, the interfered light appears, which then can be reflected by flowing blood cells, and its fluctuations can be observed by specialized sensors. The incident and reflected light are shifted in Doppler sense, which can be expressed proportionally as a velocity. The method assumes light's coherence; thus, one light splitted into two bundles is commonly used [18]. The authors found no data about the use of this method in myopic patients.

### 2.3. Retinal Vessel Analysis

Retinal vessel analysis (RVA) is a novel technique for the measurement of retinal vessel diameter in relation to time. Vessel diameter determines retinal blood flow by the change of vascular resistance. In this method, the coefficients of light reflection and absorption in retinal vessels are measured. The erythrocytes tend to absorb the light bundle with the wavelength of 400–620 nm, in opposite to the vessel tissues that mostly reflect it. The results are the local diameters of measured arteries or veins presented in UM units. The Gullstrand standard eye model is used to determine the relation of UM unit as 1 *μ*m; however, for nonemmetropic eyes, the corrective values have not been specified. One of the disadvantages of the RVA method is the necessity of the patient's pupil dilation prior to measurement. Moreover, during the measurement, patient fixations on a strobe light is required in order to provoke vascular tone changes and allow proper retinal vascular functionality analysis. The light stimulation results in percent change of baseline diameter [19].

In studies conducted by La Spina C. and company based on static and dynamic tests, the reduction of vessel diameter at the retinal posterior pole was observed in patients with high myopia. The results indicated that the functions of retinal vessels were comparable to a group of control subjects. They explained it by the reduction of oxygen consumption in the myopic group [20].

### 2.4. Retinal Function Imaging

Retinal function imaging (RFI) is the next noninvasive technique which allows to assess several haemodynamic parameters such as oximetric state, retinal blood flow velocity, capillary perfusion map, and functional metabolic responses to photic activation. The principle of this technique is multispectral, high-resolution imaging which is capable of switching up four illumination wavelengths allowing multiple image acquisition. It provides acquisition of images from small vessels, even the information about a single red blood cell in the capillary. It is also integrated with a stimulus generator which is useful for functional imaging [21]. Li and coworkers with the use of RFI found no difference in retinal microvessel blood flow velocity in myopes compared to the control group [8].

### 2.5. Laser Speckle Contrast Imaging

Aizawa and coworkers in their study observed for the first time the microcirculation within the optic disc in myopic patients, using the laser speckle contrast imaging (LSCI) method. The results have been expressed by mean blur rate coefficients, which refer to local contrast variations of the speckles and indirectly indicate the blood flow in the imaged area. The method is based on the fact that a collimated laser beam reflected from an irregular mutable surface produces random interferences, which can be observed as intensity fluctuations and inform indirectly about object movement. In order to establish this relation, an image with a longer exposure time than a speckle single fluctuation has to be captured. Thus, for selected pixels of the image, the velocity can be expressed in linear relation to contrast changes. Aizawa and coworkers noticed that eyes with myopic optic discs had decreased optic nerve microcirculation compared to the normal group [22, 23].

### 2.6. Magnetic Resonance Imaging

Magnetic resonance imaging (MRI) is the technique which uses magnetic properties of certain atomic nuclei. It provides structural and metabolic information without depth limitation. It could image blood flow by the use of intravenous contrast or by magnetically labeling blood water. The main disadvantage of this technique is low spatial resolution which prevents analysis of blood flow in different layers of the eyeball. It is sufficient to measure total blood flow and its stimuli responses in the entire retina and choroid. Blood flow of the choroid is 8–10 times higher than the retina. MRI provides quantitative value of blood flow, but there is no gold standard how to validate it. It is vulnerable to eye movements [24].

San Emeterio Nateras et al. in their study suggested that in severe myopia, blood flow is markedly reduced. Their observation was made only on two patients with severe myopia [25].

### 2.7. Optical Coherence Tomography Angiography

Optical Coherence Tomography Angiography (OCTA) is another modern noninvasive test method that does not require contrast enhancement and allows for the monitoring of the constant ocular microcirculation. It requires multiple scans of the same vessel to detect motion. This method has limited applicability and is unsuitable in eyes with nontranslucent optical centres, lack of fixation, narrow pupil, and nystagmus. Moreover, it cannot be used for peripheral retinal assessment and, unlike traditional angiography, does not allow for the visualization of vascular leakage. This device measures many parameters such as flow index, macular vessel area density, and thickness of the foveal avascular zone and central macula. The flow index estimates both vessel area and blood velocity [26]. Wang et al. used OCTA and confirmed that myopic eyes had reduced perfusion in the peripapillary retina, which may cause their increased susceptibility to vascular diseases. They also reported a negative correlation between the axial length and the peripapillary flow index [27]. Other researchers used the same method and observed decreased superficial and deep retinal vascular density in the annular zone between 0.6 and 2.5 mm from the foveal centre in myopic patients [8, 28].

### 2.8. Pulsatile Ocular Blood Flowmeter

Pulsatile Ocular Blood Flowmeter (POBF) is a well-known diagnostic technique for the evaluation of the volume of blood supplied to the retina and choroid. Measurements are taken using a pneumotonometer, which registers changes in intraocular pressure during each cardiac cycle. The results are expressed as the pulsation amplitude, reflecting the flow of blood into the eye; the volume of pulsation, that is, the amount of blood; and the frequency of pulsation, that is, the frequency of ocular flow caused by heart contraction. A study by Yang and Koh indicated decreased pulsation amplitude and blood flow volume in myopic eyes compared with emmetropic eyes [29]. Benavente-Pérez et al. and two other research groups used the same technique in young patients with myopia. They confirmed lower pulsatile ocular blood flow as well as lower ocular blood flow amplitude and volume in high myopes compared to other refractive groups [30–32].

### 2.9. Fundus Pulsation Amplitude

Tests relying on fundus pulsation amplitude (FPA) allow for the measurement of the distance between the cornea and the retina during the cardiac cycle with the use of laser interferometry. The value of this index reflects the status of the choroidal circulation. A study conducted by Berisha et al. showed a significant decrease in the FPA index along with increased axial length. Their study, however, proves experimentally that the decrease in pulsation amplitude, fundus pulsation amplitude, and pulsatile ocular blood flow in myopic eyes is related to the ocular volume and indicates that in fact, pulsatile ocular blood flow is not reduced [33].

### 2.10. Colour Doppler Imaging

The use of colour Doppler imaging (CDI) for testing ocular blood flow was first mentioned in the 1980s. This method has become popular in a very short time due to its numerous advantages, including the repeatability and reliability of measurement, availability, the low cost of testing, and suitability for nontranslucent optical structures. CDI is a noninvasive diagnostic method and is most frequently used for the analysis of retrobulbar vessels: ophthalmic artery (OA), central retinal artery (CRA), posterior ciliary arteries (PCAs) including temporal PCA (TPCA), and nasal PCA (NPCA). Testing PCAs requires a lot of experience from the operator because of their small diameter and anatomical variability. Parameters evaluated with CDI include peak systolic velocity (PSV), end-diastolic velocity (EDV), mean velocity (MV), resistance index (RI), and pulsatile index (PI). The most reliable parameters, due to their independence of the Doppler angle, are the PSV/EDV ratio and RI [30]. RI was first described by Pourcelot, and it could be calculated as RI = (PSV − EDV)/PSV. It is used for evaluating peripheral vascular resistance [34]. Vascular resistance is also related to vessel radius, vessel length, and blood viscosity, because it is consistent with the Hagen–Poiseuille law [35].

(1) $$\begin{array}{c} \Delta P=\frac{\left(8\mu LQ\right)}{\pi }R^{4}, \end{array}$$

where Δ*P* is the pressure difference between the two ends, *L* is the length of the vessel, *μ* is the dynamic viscosity, *Q* is the volumetric flow rate, and *R* is the vessel radius.

The results of the available studies using CDI indicate decreased blood flow velocity in the retrobulbar vessels correlated with greater axial length, increased refractive error, or progressive retinal degeneration, particularly in combination with chorioretinal atrophy in the peripapillary region of the optic nerve II [30, 36–41]. It should be stressed that in myopic patients, haemodynamic disorders in PCAs are associated with greater severity of degenerative fundus changes. At the same time, the extent of degenerative processes and their advancement is directly proportional to the deterioration of flow parameters in CRA [42, 43]. The most pronounced changes in blood flow have been observed in subjects with moderate and high myopia, that is, >8.0 diopters [41]. In addition, increased RI in CRA is associated with both greater axial length and refractive error, which may explain the pathomechanism of myopic retinopathy [36]. It should also be emphasized that differences in flow parameters in OA are not statistically significant with respect to retinal degeneration [42].

Doppler optical coherence tomography (DOCT) is a method which consists of a combination of optical coherence tomography with the Doppler principle. It provides high-resolution images of both static and moving components which transmits information not only about volumetry of blood flow (direction and velocity) but also about vascular anatomy. It provides three-dimensional information, and it is capable of generating a microvasculature tissue map [44]. There are almost no data about findings of DOCT in myopia. The authors found only few images which show pathological neovascularization in high myopia [45].

Retinal oximetry is the technique which measures oxygen saturation. It is based on the simultaneous capture of two images with different light wavelengths. The comparison of these images with software algorithm provides estimation of oxygen saturation, because there is a difference in light absorption by oxygenated and deoxygenated hemoglobin. [46]. Zheng and other researchers observed reduced arteriole saturation and smaller difference in arterio-venosus saturation in patients with high-grade myopia [47]. However, in a study by Yang conducted on a Chinese population, there was no significant difference between groups with small (<−3D) and moderate or high myopia (>−3D) [48].

Angiography is a gold standard for the assessment of retinal and choroidal circulation when leak is suspected. It is invasive because of the need to administer contrast agents. There are two kinds of angiography depending on the contrast agent: indocyanine green (ICG) and fluorescein angiography (FA). Fluorescein is used mainly to visualize retinal vessels whereas indocyanine allows to imagine deeper choroidal vessels. It can be used to assess blood velocity, due to measurement of the time that it takes for the dye to pass through the vessels. In myopia, Avetisov and Savitskaya found delayed blood flow with the use of fluorescein angiography [49]. It is also very helpful in degenerative myopia where it is used for evaluating development of CNV as well as RPE atrophy. ICG is better in evaluating delineation of lacquer cracks and localization of the CNV in lacquer cracks than FA in highly myopic eyes [50].

The blue field entoptic phenomenon is produced by the different light absorption by erythrocytes and leukocytes when the retina is illuminated by blue light. One could observe the motion of one's own white blood cells flowing in retinal vessels. In this method, the patient looks constantly at the screen displaying alternatingly two different images. One of them presents a solid blue surface and the other one presents an animation of moving dots. The patient's task is to select the number and minimum and maximum speeds of the generated dots in reference to one's own leukocytes; therefore, it is a subjective method. The principle of this technique is the assumption that leukocyte flux is proportional to retinal blood flow; however, it assess the blood flow only in the perimacular region [51]. The authors found no studies with the use of the above described method in myopic patients.

Measurement of ocular surface temperature (OST) becomes an important examination in modern ophthalmology. There are several known algorithms to acquire the OST that can be used for the diagnosis of ocular diseases. The thermal imaging technique, known as thermography, is the method of indirect, noninvasive measurement of body heat radiation by the use of an infrared camera. In ophthalmology, it provides patterns (images) of temperature distribution and changes in the vascular tissues within the eye and adnexa. Some studies revealed a correlation between ocular blood flow and OST. Gugleta et al. in their studies noticed a relation of corneal temperature and retrobulbar haemodynamics, where OST decreased along with the ocular circulation [52]. The authors found no studies using this method in assessment of myopic patients.

## 3. Discussion

Currently, there are many available methods for the assessment of ocular blood flow. Each one has advantages and limitations, but none of them is suitable for the direct assessment of retinal and choroidal vasculature. Each device measuring ocular blood flow presents results in different units, either relative or absolute, which makes measurements inconsistent, nonstandardized, and sometimes impossible to compare against other techniques.

Many available techniques rely on the use of laser light of various wavelengths in the process of measurement, which is a serious limitation in the case of eyes with nontranslucent optical structures or narrow pupils. CDI is the only technique relying on ultrasound waves for measurement, which makes it suitable for use in any patient, regardless of the translucency of the optical structures and visual acuity that allows for proper fixation.

In the reviewed literature, other methods of measuring tissue metabolism and providing indirect information about ocular blood flow volume were recorded, but they are not easily available. These include retinal oximetry, ocular surface temperature, and retinal functional imager.

Angiography is one of the oldest techniques but still commonly used, because of its availability and being sensitive to leakage. This method should not be performed as routine examination due to its invasive nature. Other methods are noninvasive, but some of them, for example, POBF, require physical contact of the cornea with the sensor. It may cause a risk of transmission of the infection.

The major challenge of blood flow measurement is its quantification. Only two of the described methods allow fully quantitative volumetric information. These are DOCT and MRI. Both provide information about blood flow together with structural anatomy. Nevertheless, DOCT has a higher resolution than MRI and allows assessment of blood flow with precise depth gating. Other techniques measure some of the volumetric blood flow component. Combining techniques which assess velocity with measurements of vessel diameters are possible, but these procedures are very time-consuming; therefore, they cannot be used in a clinical setting.

There are two techniques which allow to obtain high-resolution images of retinal vessels with information about blood flow: OCTA and DOCT. However, DOCT is less suitable for assessment of retinal microvasculature than OCTA. Compared to other described methods, CDI allows for the precise measurement of PSV, EDV, PI, and RI only in selected small-diameter arteries, that is, OA, PCAs, and CRA. However, due to limited resolution of the technique, microcirculation cannot be assessed. Different techniques assess various vessels; therefore, the researcher first has to decide the assessment of which vascular bed would correspond to his needs. The RVA technique has an additional advantage. Its results can be used as a predictor of the risk of cardiovascular diseases.

There are many studies focused on blood flow evaluation in myopia. In all these studies, flow parameters were significantly lower in subjects with myopia compared with subjects without refractive error. Researchers noted decreased retinal and choroidal blood flow velocities in the FPA, LDF, MRI, and CDI methods, as well as decreasing mean velocities with increasing refractive error. A similar finding was in the POBF technique which showed a lower volume of blood supplied to the retina and choroid. These observations are in line with delayed blood flow noticed in angiography. Studies with the use of the LSCI or OCTA method agreed about decreased perfusion in the peripapillary retina. In myopic eyes, there is lower retinal vascular density in the annular zone. Researchers showed also a smaller diameter of retinal vessels as well as decreased arteriole saturation compared to controls.

These differences are probably due to mechanical stretching and thinning of the tissues related to elongation of the eyeball. These changes are believed to cause a reduction in metabolic demand of tissues for oxygen and nutrients. Another theory suggests that reduced circulation leads to secondary thinning of the tissues.

## 4. Summary

Myopia is an increasingly common refractive error and a subject of interest for many researchers. The newly developed diagnostic techniques are also used for the assessment of ocular blood flow in myopia. Reduced ocular blood flow in myopia has been detected using various diagnostic techniques, and the introduction of new ones allows for more accurate measurements.

Many researchers consistently reported lower blood flow parameters in myopic eyes regardless of the used diagnostic method. It is unclear whether this is a primary change that causes secondary thinning of ocular tissues or quite the opposite; that is, the mechanical stretching of the eye wall reduces its thickness and causes a secondary lower demand of tissues for oxygen.

## Figures and Tables

**Table 1:** Table summarizing the techniques of ocular blood flow measurement in myopia.

Methods	Principle of each method	Findings in myopia
Laser Doppler flowmeter (LDF)	Measurement of blood cells traversing volume which are reflecting the light, and the light undergoes a Doppler shift	Retinal blood flow is decreased in highly and mild myopic eyes
Laser Doppler velocimetry (LDV)	Doppler shift for interfered lights reflected from blood cells	No data
Retinal vessel analyzer (RVA)	Measurement of the diameter of retinal vessels in relation to time and location	In high myopia, the vessels at the posterior pole have smaller diameter but are functionally comparable to control subjects
Retinal function imager (RFI)	Measurement of the haemodynamic parameters such as retinal blood flow velocity, oximetric state, metabolic responses to photic activation, and generation of capillary perfusion maps (CPM); RFI maps the retina to the resolution of single red blood cells moving through capillaries	No significant difference in retinal microcirculation blood flow velocity in either arterioles or venules
Laser speckle contrast imaging (LSCI)	Measurement of velocity distributions which are coded as speckle contrast variations	Decreased optic nerve microcirculation in eyes with myopic optic discs
Pulsatile ocular blood flowmeter (POBF)	Measurement with the use of a pneumotonometer, which registers changes in intraocular pressure during each cardiac cycle	In high myopes, pulsatile ocular blood flow as well as ocular blood flow amplitude and volume is decreased
Fundus pulsation amplitude (FPA)	Measurement of the distance between the cornea and the retina during the cardiac cycle with the use of laser interferometry	Decrease in the FPA index along with increased axial length
Fluorescein and indocyanine angiography (FA, ICG)	Examining the circulation of the retina and choroid using a fluorescent dye and a specialized camera	Delayed blood flow in highly myopic eyes
Optical coherence tomography angiography (OCTA)	Measurement of laser light reflectance of the surface of moving red blood cells	Reduced perfusion in the peripapillary retina and decreased superficial and deep retinal vascular density in annular zone of myopic eyes
Colour Doppler imaging (CDI)	Measurement of backscattered signals as a function of the motion of the erythrocytes toward or away from the transducer	Decreased blood flow velocity in the retrobulbar vessels, increased resistance index in central retinal artery
Doppler optical coherence tomography (DOCT)	Measuring the phase changes between two scans which is a quantitative value for the velocity if the time between the two measurements is known	DOCT could indicate choroidal neovascularization in pathological myopia
Retinal oximetry	Measurement of the optical densities of retinal vessels for two wavelengths and their ratio, which is known to be proportional to the oxygen saturation	Reduced saturation in high myopia
Magnetic resonance imaging	Method that provides structural, physiological, and functional image of tissue with the use of magnetic properties of certain atomic nuclei	In severe myopia, blood flow is markedly reduced
Blue light entoptoscopy	Measurement of average leukocyte velocity in area around the foveola by the use of entoptic phenomenon	No data
Ocular surface temperature (OST)	Measurement of infrared energy emitted from object	No data
